# Supplementary material for: Differential expression analysis of RNA sequencing data by incorporating non-exonic mapped reads
Source: BMC Genomics. 2015 Jun 11;16(Suppl 7):S14. doi: 10.1186/1471-2164-16-S7-S14 (PMC4474535; doi:10.1186/1471-2164-16-S7-S14)
Supplement: Additional file 1 — Supplementary figures and tables to provide additional analysis results. [file 1471-2164-16-S7-S14-S1.pdf]

Differential expression analysis of RNA sequencing data by incorporating non-exonic mapped reads

Hung-I Harry Chen, Yuanhang Liu, Yi Zou, Zhao Lai, Devanand Sarkar, Yufei Huang, Yidong Chen

1. Supplementary figures

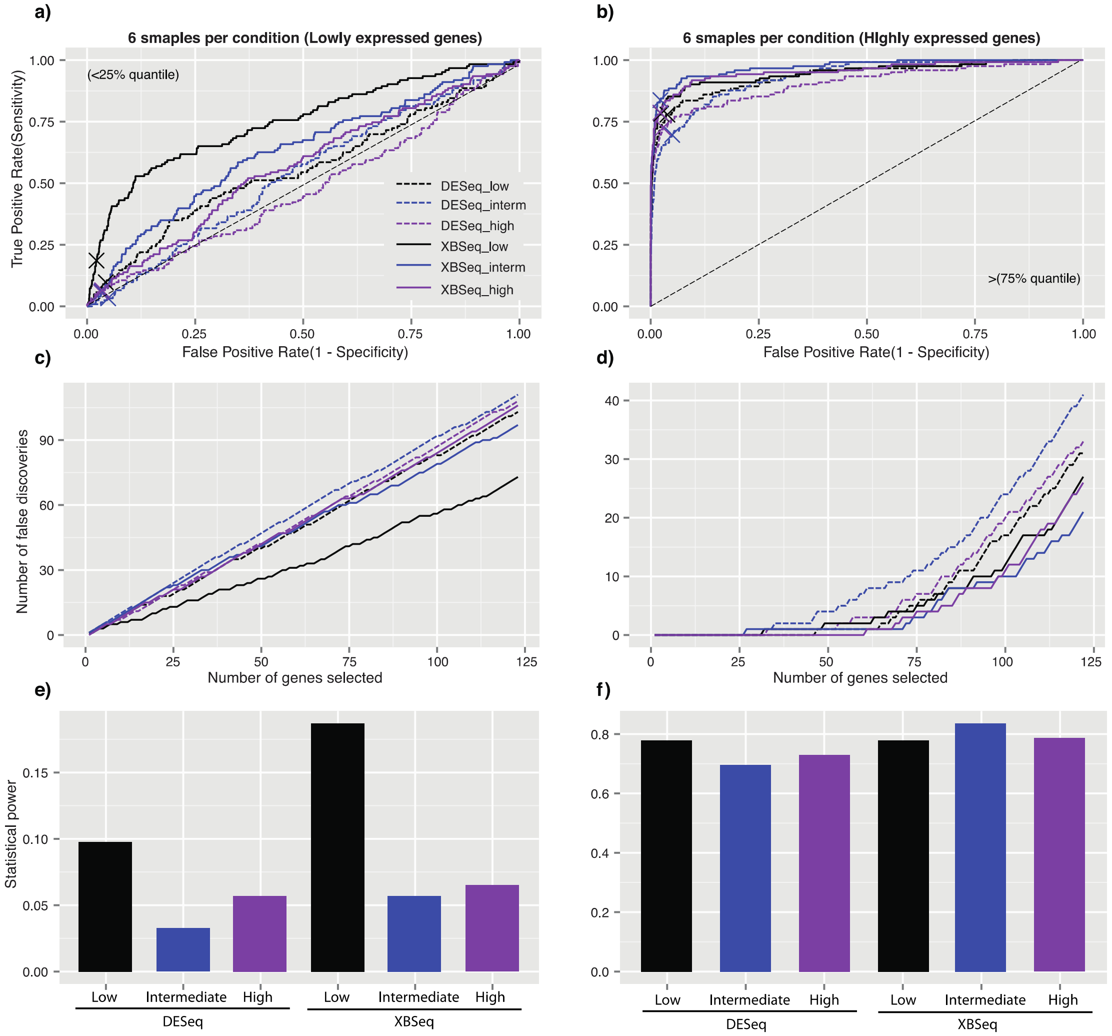

Figure S1. ROC, false discovery curve, power bar plot with highly expressed genes (>75% quantile) and lowly expressed genes (<25% quantile) with 6 number of replicates in each condition.

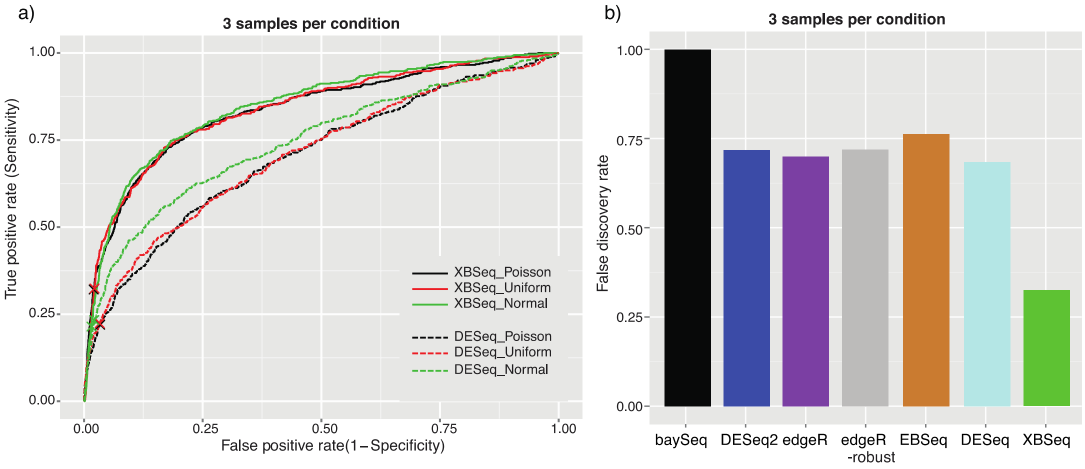

Figure S2. (a) ROC regarding simulation of background noise based on different statistical distributions, including Poisson, uniform and normal distribution. (b) False discovery rate at preset threshold with *p* value equals to 0.05.

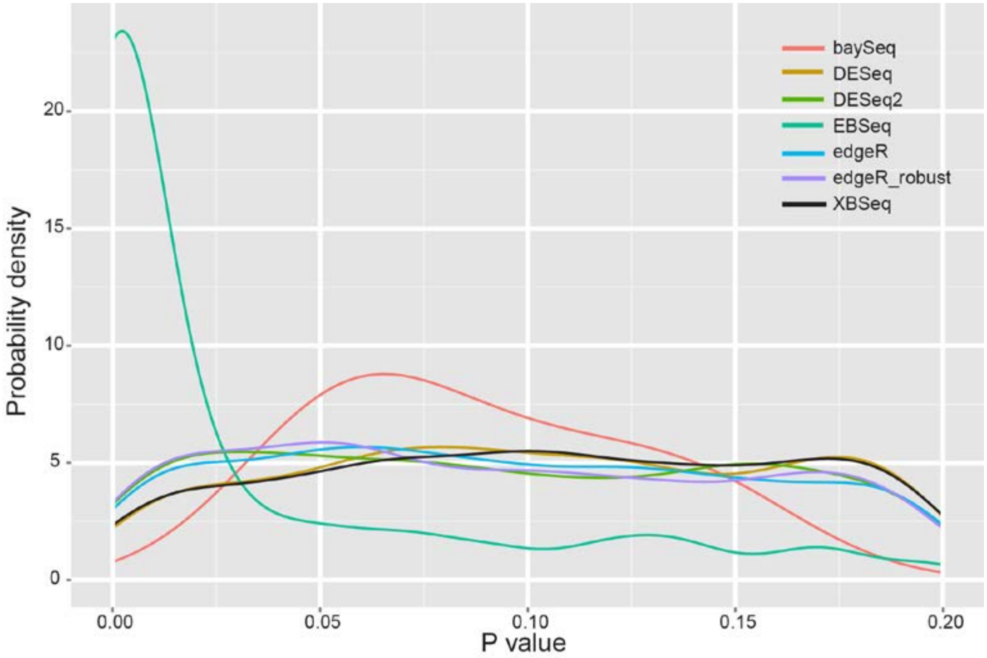

Figure S3. The *p* value distributions under null model distribution (without differentially expressed genes). Simulation was done with baseline background noise with three number of replicates.

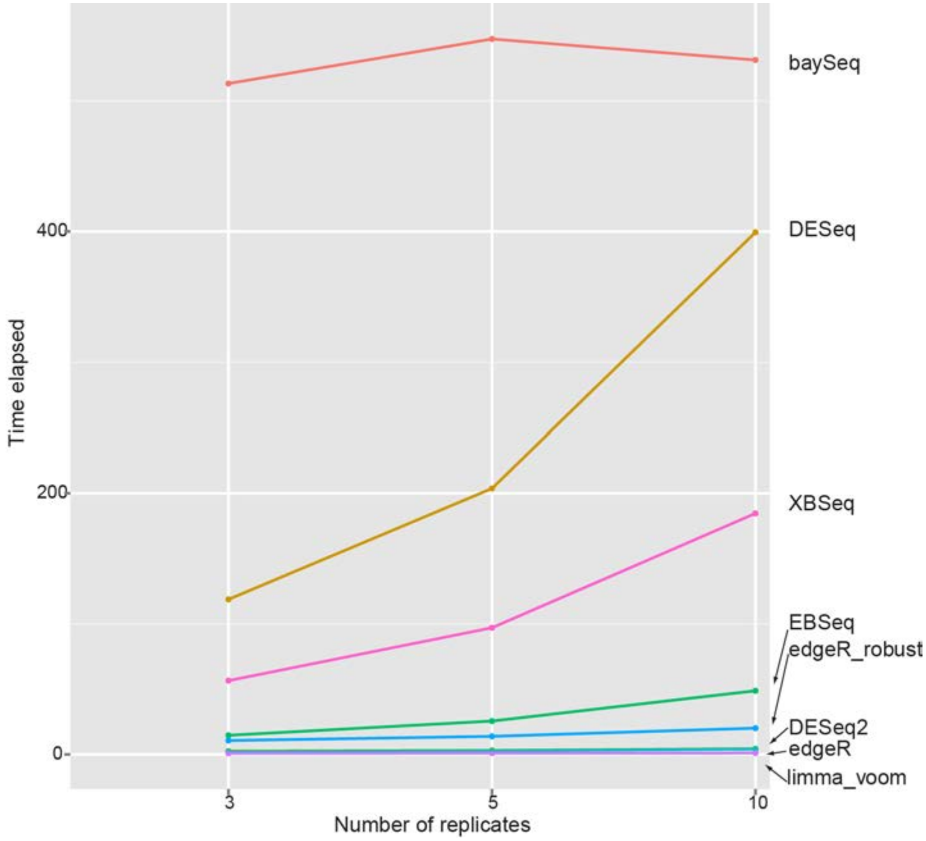

Figure S4. Execution time of different statistical methods with 3, 5, and 10 replicates in 2 conditions under high increased background noise with three number of replicates (test vs control). The comparison was performed on PC/Windows 8 system with Core i5-3570 CPU @ 3.40 GHz and 4G RAM.

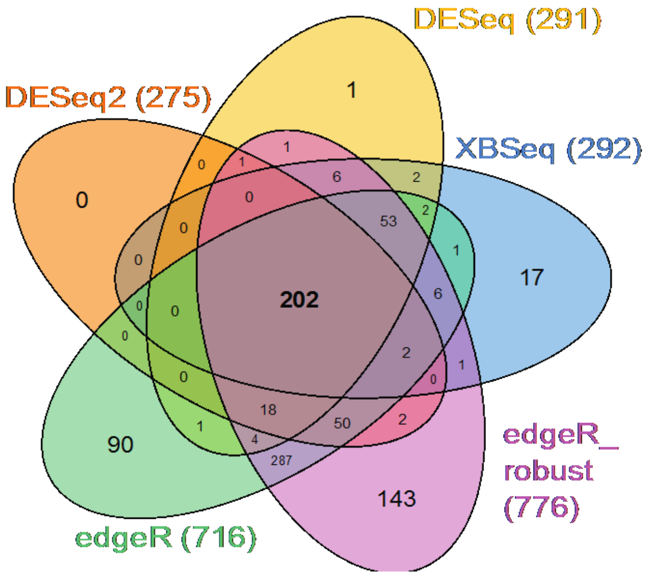

Figure S5. Venn diagram to compare the results of 5 differential gene expression analysis methods. Similar to Figure 6, XBSeq, DESeq, DESeq2, edgeR, and edgeR_robust were used to identify differential genes of real mouse RNA-seq data with p-value < 0.05, however we also require fold change > 1.5 in this analysis.

2. Supplementary Tables

	DESeq		XBSeq	
Fold change	1.5x	2x	1.5x	5x
AUC	0.89	0.98	0.89	0.98
Statistical Power	0.58	0.91	0.58	0.91

Table S1. AUC and statistical power of DESeq and XBSeq with 3 number of replicates in each condition, 10 percent of DE genes, 1.5 or 2 fold changes respectively. Each scenarios are simulated for 100 times.

		DESeq			XBSeq		
# of samples/group	Fold Change	1.5x	3x	5x	1.5fd	3x	5x
3 replicates<br>10% DEGs	AUC	0.90	1.00	1.00	0.90	1.00	1.00
	Statistical Power	0.58	0.99	1.00	0.58	0.99	1.00
3 replicates<br>30% DEGs	AUC	0.89	1.00	1.00	0.89	1.00	1.00
	Statistical Power	0.58	0.99	1.00	0.58	0.99	1.00
6 replicates<br>10% DEGs	AUC	0.96	1.00	1.00	0.97	1.00	1.00
	Statistical Power	0.84	1.00	1.00	0.85	1.00	1.00
6 replicates<br>30% DEGs	AUC	0.96	1.00	1.00	0.96	1.00	1.00
	Statistical Power	0.82	1.00	1.00	0.83	1.00	1.00

Table S2. AUC and power of DESeq and XBSeq with 3 or 6 number of replicates in each condition, 10 or 30 percent of DE genes, with 1.5, 3, or 5 fold changes respectively under baseline level of background noise.

# of samples/group		DESeq			XBSeq		
		Low	Intermediate	High	Low	Intermediate	High
3 replicates All genes	AUC	0.69	0.66	0.64	**0.82**	0.78	0.72
	Power	0.23	0.20	0.17	**0.32**	0.28	0.23
3 replicates High expression	AUC	0.79	0.78	0.77	**0.84**	0.83	0.81
	Power	0.37	0.35	0.32	**0.46**	0.43	0.41
3 replicates Low expression	AUC	0.56	0.53	0.52	**0.75**	0.66	0.57
	Power	0.07	0.05	0.05	**0.08**	0.07	0.06
6 replicates All genes	AUC	0.76	0.73	0.70	**0.89**	0.86	0.80
	Power	0.39	0.32	0.29	**0.56**	0.50	0.50
6 replicates High expression	AUC	0.94	0.93	0.91	0.95	**0.97**	0.96
	Power	0.78	0.70	0.73	0.78	**0.84**	0.79
6 replicates Low expression	AUC	0.56	0.54	0.47	**0.75**	0.63	0.57
	Power	0.10	0.03	0.06	**0.19**	0.06	0.07

Table S3. AUC and power of DESeq and XBSeq with 3 or 6 number of replicates in each condition, 10 percent of DE genes, 1.5 fold change with low, intermediate or high level of non-exonic mapped reads respectively.

3 replicates/group	**baySeq**	**DESeq2**	**edgeR**	**edgeR -robust**	**Limma**	**EBSeq**	**DESeq**	**XBSeq**
AUC	0.63	0.65	0.65	0.65	0.65	0.65	0.65	**0.73**
Statistical Power	0	0.22	0.23	**0.25**	0.00	0.09	0.18	0.24
6 replicates/group								
AUC	0.70	0.71	0.71	0.70	0.71	0.71	0.71	**0.80**
Statistical Power	0.02	0.33	0.34	**0.37**	0.04	0.07	0.30	0.33

Table S4. AUC and power of eight different statistical methods with 3 or 6 number of replicates in each condition, 10 percent of DE genes, 1.5 fold change with high level of non-exnoic mapped reads respectively. The best result is in bold face.

Cutoff Criterion	XBSeq	DESeq	DESeq2	edgeR	edgeR_robust
P<0.05	446	414 (91.6%)	421 (40.8%)	402 (44.2%)	429 (40%)
P<0.05 & FC > 1.5	292	265 (91.1%)	204 (74.2%)	266 (37.2%)	270 (34.8%)

Table S5. Number of overlapped DE genes detected by different algorithms comparing with XBSeq result in two different cutoff criterions.
